# HALP, PIV, and SII as novel composite inflammatory indices for early detection and severity assessment of Alzheimer’s disease

**DOI:** 10.3389/fnagi.2025.1711176

**Published:** 2025-12-15

**Authors:** Chao Huang, Chenxi Lu, Shuai Liu, Fanshu Dai, Dilraba Mahmut, Hezhen Gao, Yong Ji, Biao Zhang

**Affiliations:** 1 Department of Clinical Laboratory, Huanhu Hospital Affiliated to Tianjin Medical University, Tianjin, China; 2 Department of Neurology, Huanhu Hospital Affiliated to Tianjin Medical University, Tianjin, China; 3 Tianjin Key Laboratory of Cerebral Vascular and Neurodegenerative Diseases, Tianjin, China

**Keywords:** Alzheimer’s disease, biomarkers, dementia severity, inflammatory indices, neuroinflammation

## Abstract

**Objective:**

This study aimed to evaluate the diagnostic and prognostic value of the Hemoglobin, Albumin, Lymphocyte, and Platelet (HALP) Score, the Pan-Immune-Inflammation Value (PIV), and the Systemic-Immune-Inflammation Index (SII) in Alzheimer’s disease (AD), exploring their association with dementia severity and their potential utility in diagnosis and monitoring disease progression.

**Methods:**

In a retrospective case-control study, 261 AD patients and 176 healthy controls were enrolled. Propensity score matching (PSM) generated a balanced cohort of 176 patient-control pairs. Demographic, clinical, and hematologic variables were collected, including HALP, PIV, and SII, and dementia severity was assessed using the mini-mental state examination (MMSE). Univariate and multivariate logistic regression analyses were performed to identify independent risk factors for AD, while spearman’s correlation and receiver operating characteristic (ROC) curve analysis with bootstrap internal validation were used to evaluate the biomarker’s performance.

**Results:**

Following matching, AD patients exhibited significantly lower HALP and higher PIV and SII levels indicating a chronic pro-inflammatory state. HALP, PIV, and SII showed gradual but non-significant changes with dementia severity. HALP exhibited inverse correlation trend with dementia severity, though it did not reach statistical significance. Logistic regression identified education level and elevated neutrophil counts as independent risk factors of AD. ROC analysis revealed modest diagnostic performance for indices (AUC from 0.627 to 0.655), while combination of them did not significantly improve the diagnostic power.

**Conclusion:**

HALP, PIV, and SII are promising blood-based biomarkers for AD diagnosis and progression monitoring. HALP may help track disease progression. These low cost, accessible composite inflammatory indices offer potential as adjunct tools for early detection and severity assessment in AD, especially in resource limited settings.

## Introduction

Alzheimer’s disease (AD), the most common neurodegenerative disorder and leading cause of dementia, presents a mounting public health crisis, with projections indicating 152 million affected individuals by 2050 (Livingston et al., 2020). Characterized by progressive cognitive decline and impairments in memory, reasoning, and daily functioning, AD remains difficult to diagnose early due to the absence of definitive biomarkers and reliance on costly neuroimaging or invasive cerebrospinal fluid analysis (Palmqvist et al., 2020). Consequently, there is an urgent need for accessible peripheral biomarkers that reflect underlying pathophysiological processes, particularly neuroinflammation, a critical AD driver that accelerates neurodegeneration independently of classical amyloid-β plaques and tau tangles (Heneka et al., 2015).

Emerging evidence implicates systemic inflammation in AD pathogenesis through brain-periphery crosstalk (Bettcher et al., 2021; Huang et al., 2021; Tao et al., 2018), where neutrophils, lymphocytes, and platelets modulate neurovascular integrity and blood–brain barrier permeability (Prinz and Priller, 2017). This has spurred interest in hematologic indices like the neutrophil to lymphocyte ratio (NLR) and platelet to lymphocyte ratio (PLR). However, these metrics demonstrate inconsistent AD associations across cohorts and fail to capture multidimensional immune nutritional interactions (Szczechowiak et al., 2019). Recent research highlights peripheral blood markers as cost effective, easily measurable tools for early detection and stratification of dementia severity in AD. However, conventional indices often show inconsistent associations and fail to reflect complex immune-nutritional interactions. Novel composite indices such as the Hemoglobin, Albumin, Lymphocyte, and Platelet score (HALP), the Pan-Immune-Inflammation Value (PIV), and the Systemic-Immune-Inflammation Index (SII) offer broader insights, HALP reflects nutritional immune balance, PIV quantifies innate-adaptive immune dynamics, and SII assesses thromboinflammatory activity (Kından et al., 2025; Okyar Baş et al., 2023; Xia et al., 2023), yet their application in AD remains largely unexplored.

While prior population-based studies have established associations between systemic inflammatory indices and cognitive performance (Guo et al., 2024), their utility for the diagnosis and severity stratification of clinically confirmed AD patients remains unexplored. Therefore, this study aims to not only compare these novel indices, including HALP, PIV and SII, between a rigorously defined AD cohort and healthy controls but also to examine their associations with clinically assessed dementia severity and evaluate their diagnostic and stratification performance, both individually and in combination, using receiver operating characteristic (ROC) curve analysis.

## Materials and methods

### Study design

This was a retrospective case-control study conducted at Huanhu Hospital Affiliated to Tianjin Medical University. This study protocol was approved by Tianjin Huanhu Hospital Ethics Committee (Approval Number 2019-40). Informed consent was waived due to the retrospective nature of the study, and all procedures adhered to the ethical principles of the Declaration of Helsinki. This study was reported in accordance with STROBE guidelines for case-control studies. The completed STROBE checklist is available as Supplementary material.

### Participants

A total of 261 AD patients and 176 healthy controls were initially identified by a retrospective screening of hospital records from September 2019 to January 2024. The inclusion criteria for AD patients were a clinical diagnosis of AD based on the National Institute on Aging and the Alzheimer’s Association (NIA-AA) criteria and age ≥ 60 years. Exclusion criteria included: (1) History of stroke; (2) Other neurological conditions causing cognitive decline such as Parkinson’s disease or frontotemporal dementia; (3) Other systemic diseases causing cognitive decline such as thyroid dysfunction, severe anemia, syphilis, HIV; (4) History of psychosis or intellectual disability; and (5) Cognitive decline attributed to traumatic brain injury. Healthy controls were selected from cognitively healthy individuals who underwent a comprehensive medical evaluation and were matched by age and gender to the AD group.

### Demographic and clinical data collection

General demographic characteristics of patients and controls were collected from the hospital information system, including gender, age, education years, hypertension, diabetes, dyslipidemia and Mini-Mental State Examination (MMSE) scores which were through structured interviews and clinical evaluations.

### Assessment of dementia severity

Dementia severity was categorized using the MMSE score. Based on this score, the patients in our cohort were grouped into mild (MMSE 21–26), moderate (MMSE 10–20), and severe dementia (MMSE ≤ 10) groups. This classification was corroborated with neuroimaging and clinical evaluations performed by an experienced neurologist.

### Laboratory examination

Fasting elbow venous blood samples were collected from all participants in the morning after at least 8 h of fasting. Blood samples were processed within 30 min of collection. For biochemical tests, blood was collected into separation gel tubes. For blood routine test, blood was collected into heparin anticoagulant tubes. Albumin (Alb) levels were measured using an automated biochemical analyzer (AU5800 automatic biochemistry analyzer, Beckman). Hemoglobin (HGB), Neutrophil (Neu), Lymphocyte (LYM), Monocyte (Mono), and Platelet (PLT) counts were determined through a complete blood count (CBC) using an automated hematology analyzer (XN-1000 analyzer, Sysmex). The novel composite inflammatory indices were calculated as follows:


$$\text{HALP = }\frac{hemoglobin\times albumin\times lymphocytecount}{plateletcount}\left(Yalav\;etal.,\;2021\right)$$

$$\text{PIV}=\frac{n\,e\,u\,t\,r\,o\,p\,h\,i\,l\,c\,o\,u\,n\,t\times m\,o\,n\,o\,c\,y\,t\,e\,c\,o\,u\,n\,t\times p\,l\,a\,t\,e\,l\,e\,t\,c\,o\,u\,n\,t}{l\,y\,m\,p\,h\,o\,c\,y\,t\,e\,c\,o\,u\,n\,t}\left(Li\;etal.,\;2025\right)$$

$$\text{SII}=\frac{n\,e\,u\,t\,r\,o\,p\,h\,i\,l\,c\,o\,u\,n\,t\times p\,l\,a\,t\,e\,l\,e\,t\,c\,o\,u\,n\,t}{l\,y\,m\,p\,h\,o\,c\,y\,t\,e\,c\,o\,u\,n\,t}\left(Xu\;etal.,\,2021\right)$$

### Statistical analysis

All statistical analyses were performed using SPSS software (version 29.0) and R software (version 4.5.1). Continuous variables were expressed as mean ± standard deviation (SD) or median and interquartile range (IQR) depending on their distribution, while categorical variables were expressed as frequency and percentages. To ensure data completeness, we first assessed the dataset for missing values. No missing data was identified for any of the demographic, clinical, or laboratory variables used in the primary analyses, hence, no specific imputation methods were required. To address potential confounding and enhance the comparability between AD patients’ group and healthy controls group, we performed propensity score matching (PSM). Cases and controls were matched 1:1 using the nearest-neighbor algorithm without replacement, with a caliper width of 0.02 of the standard deviation of the logit of the propensity score. Matching was performed based on age and sex. The balance of covariates before and after matching was assessed using standardized mean differences (SMD) with an SMD < 0.10 indicating a good balance. All subsequent analyses were primarily conducted on this PSM cohort.

Group comparisons employed Student’s *t*-test or ANOVA for normally distributed continuous variables, and the Mann-Whitney U test or Kruskal-Wallis test were used for the variables following non-normal distribution. The chi-square test or Fisher’s exact test for categorical variables. Univariate logistic regression analysis was conducted to assess the association between demographic, clinical, and biochemical variables with the presence of AD. Multivariate logistic regression was used to determine independent risk factors for AD, adjusting for potential confounders. Odds ratios (ORs) with their 95% confidence intervals (CIs) were reported. Spearman’s rank correlation was used to assess the relationship between HALP, PIV, and SII with dementia severity, with correlation coefficients and their 95% CIs reported.

The diagnostic performance of biomarkers, including HALP, PIV, and SII, was evaluated using ROC curve analysis. The area under the curve (AUC) with its 95% CIs was calculated. To assess the stability and optimism of the ROC model, internal validation was performed using bootstrapping with 1,000 resamples. The bootstrap corrected AUC and its 95% CIs were reported for the primary combined model. positive predictive value (PPV), and negative predictive value (NPV) were calculated.

As an exploratory retrospective study, a prior sample size calculation was not feasible, while, to assess the statistical power of our final sample, a *post-hoc* power analysis was conducted using G Power software (version 3.1.9) for the primary logistic regression model. Based on the observed effect sizes in PSM cohort (*n* = 352), with an α level of 0.05, the analysis indicated that our study achieved a statistical power of over 80% to detect the significant associations reported. A two tailed *P-*value of < 0.05 was considered statistically significant.

## Results

### Comparison of demographic and hematological characteristics before and after propensity score matching in recruited participants

To enhance the robustness of our comparisons, we performed propensity score matching (PSM) on age and sex, resulting in a well-balanced cohort of 176 AD patients and 176 healthy controls. As shown in Table 1, the standardized mean differences (SMDs) for most covariates were reduced, although a residual imbalance persisted. All subsequent analyses are based on this matched cohort.

**TABLE 1:** Baseline characteristics of Alzheimer’s disease patients and healthy controls before and after propensity score matching (PSM).

Variables	Before PSM				After PSM			
	Controls (*n* = 176)	Patients with AD (*n* = 261)	*P*	SMD	Controls (*n* = 176)	Patients with AD (*n* = 176)	*P*	SMD
Gender, n(%)			0.779	0.027			1.000	< 0.001
Male	84(47.7%)	121(46.4%)			84(47.7%)	84(47.7%)		
Female	92(52.3%)	140(53.6%)			92(52.3%)	92(52.3%)		
Age, years	71(65, 77)	73(67, 78)	0.456	0.072	71(65, 77)	72(67, 77)	0.925	0.012
Education, years	14(14, 16)	9(5, 12)	< 0.001*	1.586	14(14, 16)	10(6, 12)	< 0.001*	1.218
Hypertension, n (%)	57(32.4%)	96(36.8%)	0.345	0.093	57(32.4%)	61(34.7%)	0.735	0.048
Diabetes, n (%)	20(11.4%)	45(17.2%)	0.09	0.168	20(11.4%)	28(15.9%)	0.277	0.133
Dyslipidemia, n (%)	14(8%)	34(13.0%)	0.096	0.166	14(8%)	20(11.4%)	0.367	0.116
Alb, g/L	44.70(42.70, 46.20)	43.30(41.40, 45.45)	< 0.001*	0.534	44.70(42.70, 46.20)	42.45(40.48, 44.40)	< 0.001*	0.506
HGB, g/L	141.0(132.0, 152.0)	138.0(129.00, 148.00)	0.002*	0.319	141.0(132.0, 152.0)	138.0(126.0, 147.25)	0.008*	0.300
Neu, 10^9^/L	3.32(2.69, 4.03)	3.59(2.88, 4.52)	< 0.001*	0.425	3.32(2.69, 4.03)	3.74(3.11, 4.48)	< 0.001*	0.396
LYM, 10^9^/L	2.02(1.54, 2.61)	1.78(1.41, 2.22)	< 0.001*	0.162	2.02(1.54, 2.61)	1.67(1.32, 2.06)	< 0.001*	0.161
Mono, 10^9^/L	0.34(0.28, 0.43)	0.34(0.28, 0.43)	0.699	0.065	0.34(0.28, 0.43)	0.35(0.28, 0.43)	0.915	0.024
PLT,10^9^/L	238.0(199.0, 267.0)	226.0(191.0, 262.0)	0.007*	0.262	238.0(199.0, 267.0)	222.5(189.75, 256.00)	0.019*	0.267
HALP	54.38(40.06, 68.03)	47.89(35.26, 61.57)	< 0.001*	0.151	54.38(40.06, 68.03)	45.21(33.51, 55.94)	< 0.001*	0.149
PIV	127.85(89.13, 187.87)	159.50(102.89, 235.41)	< 0.001*	0.344	127.85(89.13, 187.87)	178.92(119.23, 266.85)	< 0.001*	0.308
SII	382.54(271.05, 549.45)	454.31(325.65, 631.49)	< 0.001*	0.503	382.54(271.05, 549.45)	506.48(363.18, 658.42)	< 0.001*	0.495

*P < 0.05. SMD, standardized mean differences.

In the matched cohort, AD patients showed significant peripheral nutritional and inflammatory dysregulation compared with controls, characterized by significantly lower HALP and higher PIV and SII (Table 1). In addition, AD patients had lower albumin and hemoglobin concentrations, suggesting potential nutritional deficits or underlying systemic inflammation. indicating their potential as accessible blood-based biomarkers that reflect the inflammatory and nutritional imbalance central to AD pathophysiology. Moreover, analysis of cellular components revealed immune activation, characterized by elevated neutrophil counts and reduced lymphocyte counts. Collectively, these findings support the view that AD is associated with a chronic pro-inflammatory state.

### Progressive impairment of cognitive reserve and nutritional inflammatory status across dementia severity stages

As delineated in Table 2, which includes data from the matched AD patient’s cohort, with advancing dementia severity, patients showed both a progressive reduction in cognitive reserve (education years). Education level showed a significantly decreasing trend with disease progression, consistent with cognitive reserve theory. HALP levels displayed a descending trend from mild to moderate and severe dementia, however, PIV and SII showed no significant difference across the dementia stages. Despite lack of significance, the HALP descending trends suggest that HALP, rather than PIV and SII, might better capture subtle shifts in systemic inflammation and nutritional status along disease progression, warranting further validation in larger, longitudinal cohorts.

**TABLE 2:** Severity dependent changes in hematologic and novel composite inflammatory biomarkers across dementia stages in the propensity score matched Alzheimer’s disease cohort.

Variables	Mild dementia (*n* = 56)	Moderate dementia (*n* = 94)	Severe dementia (*n* = 26)	*P*	*P*		
					Mild vs. moderate	Mild vs. severe	Moderate vs. severe
Gender, n(%)				0.764	0.759	0.026*	0.037*
Male	26(46.4%)	47(50.0%)	11(42.3%)				
Female	30(53.6%)	47(50.0%)	15(57.7%)				
Age, years	72.00(65.00, 78.00)	72.00(65.00, 77.00)	71.00(64.00, 78.00)	0.991			
Education, years	10(9, 12)	9(8, 12)	8(6, 11)	0.032			
Hypertension, n (%)	22(39.3%)	31(33.0%)	8(30.7%)	0.664			
Diabetes, n (%)	11(19.6%)	13(13.8%)	4(15.4%)	0.640			
Dyslipidemia, n (%)	7(12.5%)	12(12.8%)	1(3.8%)	0.424			
Alb, g/L	42.40(41.08, 44.18)	42.45(39.90, 44.53)	42.65(41.20, 44.43)	0.677			
HGB, g/L	138.00(126.75, 146.75)	139.00(126.75, 149.00)	130.00(122.00, 141.00)	0.211			
Neu, 10^9^/L	4.14(3.23, 4.81)	3.63(2.83, 4.69)	3.61(3.08, 4.56)	0.297			
LYM, 10^9^/L	1.82(1.31, 2.17)	1.67(1.39, 2.05)	1.46(1.25, 1.75)	0.105			
Mono, 10^9^/L	0.38(0.28, 0.43)	0.34(0.28, 0.42)	0.31(0.26, 0.48)	0.730			
PLT,10^9^/L	223.50(189.00, 259.75)	221.00(191.00, 248.50)	229.50(174.75, 258.75)	0.830			
HALP	48.28(34.93, 63.15)	45.21(33.73, 57.05)	39.04(30.67, 49.53)	0.116			
PIV	181.42(123.43, 304.08)	172.49(116.16, 228.18)	182.61(121.44, 331.15)	0.498			
SII	507.42(377.58, 691.00)	502.68(358.35, 641.36)	577.83(348.66, 696.57)	0.542			

*P < 0.05.

### Logistic regression analyses identified key predictors of Alzheimer’s disease

Based on PSM cohort, logistic regression analyses identified key predictors of AD (Table 3). Univariate logistic regression analysis revealed that increased neutrophil counts and elevated novel composite inflammatory indices (PIV, SII) were associated with greater AD risk, while higher education years, serum albumin, hemoglobin, and HALP were protective factors. All results are reported as odds ratios (OR) with 95% confidence intervals (CI). In the multivariate model, lower education and elevated neutrophil counts remained independent predictors of AD. Notably, although HALP, PIV, and SII showed significant in univariate logistic regression, their associations with AD lost statistical significance after adjustment for key demographic and clinical factors in multivariate model.

**TABLE 3:** Univariate and multivariate logistic regression analysis of Alzheimer’s disease risk factors in the propensity score matched cohort.

Variables	Univariate analysis		Multivariate analysis	
	OR(95% CI)	*P*	OR(95% CI)	*P*
Gender	1.000(0.658–1.519)	1.000		
Age, years	1.021(0.998–1.045)	0.893		
Education, years	0.592(0.523–0.669)	< 0.001	0.593(0.518–0.679)	< 0.001*
Hypertension, n (%)	1.107(0.711–1.724)	0.652		
Diabetes, n (%)	1.476(0.797–2.733)	0.216		
Dyslipidemia, n (%)	1.484(0.724–3.040)	0.281		
Alb, g/L	0.837(0.776–0.902)	< 0.001	0.905(0.812–1.009)	0.071
HGB, g/L	0.980(0.966–0.994)	0.006	0.997(0.973–1.022)	0.801
Neu, 10^9^/L	1.417(1.171–1.714)	< 0.001	1.771(1.121–2.799)	0.014*
LYM, 10^9^/L	0.383(0.265–0.554)	< 0.001	0.431(0.096–1.941)	0.273
Mono, 10^9^/L	1.215(0.226–6.533)	0.820		
PLT,10^9^/L	0.995(0.992–0.999)	0.014	0.993(0.981–1.006)	0.305
HALP	0.977(0.966–0.987)	< 0.001	0.990(0.953–1.029)	0.605
PIV	1.003(1.001–1.005)	< 0.001	1.000(0.997–1.004)	0.862
SII	1.002(1.001–1.003)	< 0.001	1.000(0.996–1.003)	0.840

*P < 0.05.

### Correlation of HALP, PIV, and SII with dementia severity

In the PSM cohort, Spearman’s correlation analysis revealed that the inverse relationship between HALP and dementia severity did not reach statistical significance, while there persisted as a strong non-significant trend (*r* = –0.138, *P* = 0.067) (Table 4). This suggests that the association between lower HALP levels and more advanced cognitive impairment is robust to adjustment for age and sex, though the effect may be more modest than indicated by the unadjusted analysis. In contrast, neither PIV nor SII showed any significant linear correlation with dementia severity scores in the matched cohort.

**TABLE 4:** Correlations of HALP, PIV, and SII with dementia severity in the propensity score matched cohort.

Variables	HALP		PIV		SII	
	r (95% CI)	*P*	r (95% CI)	*P*	r (95% CI)	*P*
Dementia severity	–0.138(–0.281–0.010)	0.067	–0.021(–0.168–0.128)	0.786	0.003(–0.145–0.151)	0.966

### ROC curve analysis of HALP, PIV, and SII for discriminating Alzheimer’s disease from healthy controls

**FIGURE 1:**
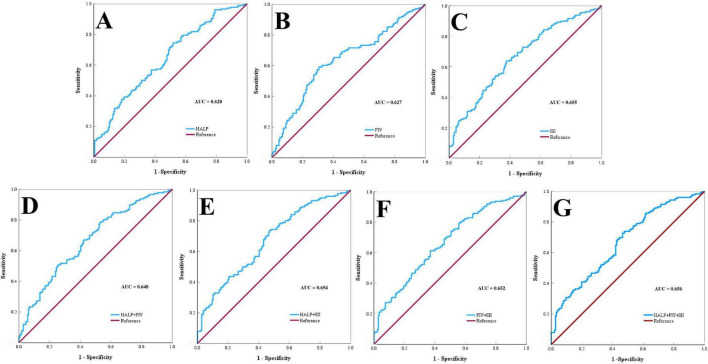
Receiver operating characteristic (ROC) curves of inflammatory indices for discriminating against AD in the propensity score matched cohort. (A–C) The ROC curves for individual indices: HALP, PIV, and SII. (D–F) The diagnostic efficacy of pairwise combinations HALP+PIV, HALP+SII, and PIV+SII, respectively. (G) The ROC curve of the composite model combining HALP+PIV+ SII.

ROC curve analysis was used to evaluate the ability of HALP, PIV and SII to differentiate AD patients from healthy controls in the matched cohort. All indices demonstrated statistically significant but modest discriminative power (Table 5; Figure 1). Among individual markers, SII achieved the highest area under the curve (AUC) of 0.655 (95% CI: 0.598–0.712), with a sensitivity of 0.636 and specificity of 0.625, HALP and PIV each yielded similar AUC values (0.628 and 0.627, respectively). However, HALP showed higher sensitivity (0.733), while PIV exhibited higher specificity (0.693). Notably, biomarker combinations did not yield diagnostic improvements.

**TABLE 5:** Diagnostic performance of HALP, PIV and SII for discriminating Alzheimer’s disease from healthy controls in the propensity score matched cohort.

Variables	Sensitivity (95% CI)	Specificity (95% CI)	AUC (95% CI)	Bootstrap corrected AUC (95% CI)	PPV	NPV	Youden index	*P*
HALP	0.733 (0.664–0.794)	0.500 (0.426–0.576)	0.628 (0.570–0.686)	0.629 (0.572–0.685)	0.595	0.652	0.233	< 0.001*
PIV	0.540 (0.465–0.614)	0.693 (0.621–0.758)	0.627 (0.569–0.685)	0.625 (0.567–0.680)	0.636	0.601	0.233	< 0.001*
SII	0.636 (0.562–0.706)	0.625 (0.550–0.696)	0.655 (0.598–0.712)	0.656 (0.599–0.711)	0.629	0.632	0.261	< 0.001*
HALP+PIV	0.784 (0.718–0.814)	0.466 (0.391–0.541)	0.648 (0.591–0.705)	0.649 (0.591–0.703)	0.595	0.683	0.250	< 0.001*
HALP+SII	0.711 (0.651–0.766)	0.477 (0.394–0.544)	0.654 (0.597–0.710)	0.654 (0.595–0.706)	0.603	0.689	0.261	< 0.001*
PIV+SII	0.614 (0.538–0.685)	0.631 (0.554–0.700)	0.652 (0.596–0.709)	0.653 (0.597–0.710)	0.624	0.620	0.244	< 0.001*
HALP+PIV+SII	0.778 (0.710–0.837)	0.472 (0.394–0.545)	0.651 (0.595–0.708)	0.650 (0.593–0.705)	0.596	0.686	0.250	< 0.001*

*P < 0.01.

Crucially, to assess the stability and generalizability of our primary model, we performed internal validation using bootstrapping with 1,000 resamples. The bootstrap corrected AUC and its 95% CI are reported in Table 5. Bootstrap-corrected AUC values were nearly identical to the original estimates across all models. All individual markers and combinations showed statistically significant discriminatory power, with *P* < 0.001 in each case.

## Discussion

This study systematically investigated novel composite inflammatory indices (HALP, PIV, and SII) in relation to AD diagnosis and severity. A key strength of our analysis is the use of a rigorously matched case-control cohort, achieved through propensity score matching on age and sex, which ensured excellent baseline comparability. This methodological rigor, combined with internal bootstrap validation, strengthens the evidence that systemic inflammation and nutritional status are intricately linked with AD pathophysiology and provides a robust foundation for our findings.

Our work provides critical advancements beyond large population-based studies like the NHANES, which established a correlation between systemic inflammatory indices (including SII and PIV) and cognitive performance scores in the general elderly population (Guo et al., 2024). First, we focused specifically on a well-defined clinical case-control cohort, allowing us to evaluate the direct utility of these indices for distinguishing clinically diagnosed AD patients from healthy controls, rather than predicting cognitive scores across a population continuum. Second, we introduced and comprehensively evaluated the HALP score, a novel composite index that uniquely integrates nutritional status with inflammatory status, which was not examined in the NHANES study. Third, by employing a case-control design reinforced with PSM, we enhanced the comparability between groups and the robustness of our associations. Finally, we moved beyond reporting simple association by rigorously assessing the diagnostic accuracy and stability of these indices using ROC analysis with bootstrap internal validation, providing a more reliable estimate of their potential clinical performance.

Consistent with the cognitive reserve hypothesis, education level remained a strong independent protective factor (Meng and D’Arcy, 2012). Elevated neutrophil count emerged as an independent risk factor, aligning with prior studies showing peripheral neutrophilia and NLRP3 (NOD-, LPR- and pyrin domain-containing protein 3) inflammasome activation contribute to AD pathology (Jose et al., 2022; Xu et al., 2025). This finding supports the hypothesis that systemic inflammation may exacerbate central neuroinflammatory responses.

Although HALP lost significance in multivariate regression, it retained an inverse correlation trend with dementia severity. This suggests HALP captures broader aspects of disease progression, combining markers of malnutrition (albumin, hemoglobin) and inflammation (lymphocyte and platelet counts). Prior studies reported similar associations between lower HALP and more advanced cognitive impairment (Gürbüzer and Ozkaya, 2024). In contrast, PIV and SII significantly elevated in AD patients, while non-significant correlations with severity. These indices primarily reflect systemic immune activation, characterized by increased neutrophils and platelets with reduced lymphocytes, previously linked to cognitive decline (Aries and Hensley-McBain, 2023; Li et al., 2023; Zenaro et al., 2015).

ROC analysis demonstrated that single blood based composite inflammatory indices (HALP, PIV, and SII) each provided statistically significant modest discriminatory power in distinguishing AD patients from healthy controls within our matched cohort. This underscored their potential role as accessible screening aids. Notably, contrary to our initial hypothesis, the combination of these indices did not yield a statistically significant improvement in diagnostic accuracy over the best performing single marker. The stability and generalizability of this finding were confirmed through bootstrap internal validation, which provided a robust, corrected estimate of the model’s performance. Consequently, our findings refine the inflammation AD paradigm (Yang et al., 2025), suggesting that while these systemic inflammatory indices are independently associated with AD presence, their combined use does not appear to offer a substantial diagnostic advantage. Therefore, HALP, PIV and SII may serve best as cost effective, initial triage tools that could complement, rather than integrate with, established core biomarkers like CSFAβ42, tau, and plasma p-tau for risk stratification in resource conscious settings (Sancesario and Bernardini, 2019). Elevated neutrophil counts predictive power highlights systemic inflammation as a potential therapeutic target (Calsolaro and Edison, 2016).

Our study has limitations. First, its single center, retrospective nature means that, despite the use of PSM to enhance internal validity and a *post-hoc* power analysis confirming adequate power for our primary findings, the sample size may still be limited, and the results require external validation in larger, multicenter, prospective cohorts. Second, the lack of direct correlation with established CSF or neuroimaging biomarkers limits the mechanistic interpretation of our findings. Third, the sample size was not based on a prospective power calculation, which is a common limitation in retrospective studies due to retrospective study utilizing all available data with a defined period. While our *post-hoc* power analysis confirmed adequate power (>80%) for our primary findings and we employed PSM to enhance robustness, future prospective studies with pre-specified sample sizes are warranted to confirm these results. Longitudinal studies are needed to assess the temporal dynamics of these markers. Integrating them into multi-modals with plasma p-tau, glial fibrillary acidic protein (GFAP), or neuroimaging may enhance diagnostic precision. Investigating treatment responses such as anti-inflammatory interventions could establish clinical utility.

In conclusion, our case-control study, conducted within a rigorously matched cohort, demonstrates that blood based composite inflammatory indices, particularly HALP, PIV and SII, are significantly associated with Alzheimer’s disease. A key finding is that individual blood based inflammatory indices showed modest yet significant power in discriminating against AD from healthy controls, while the combination of these indices did not confer a significant advantage over individual markers for cross sectional disease identification. The stability of this finding was confirmed through internal bootstrap validation. Therefore, these readily accessible and cost-effective indices are best positioned as independent adjuncts for initial patient stratification and risk assessment, rather than as an integrated diagnostic panel. Their integration into routine laboratory workups could enhance the management of AD, especially in resource limited settings. Future large scale, prospective studies are essential to validate these findings and to elucidate the precise mechanism linking systemic immunonutritional status to AD pathogenesis.

## Data Availability

The datasets presented in this article are not readily available because data is confidential and cannot be shared publicly. Requests to access the datasets should be directed to zhangbiao0902@aliyun.com.
